# Slice-Level Deep Learning Classification of Acute Cholecystitis on Contrast-Enhanced CT: A Single-Center Benchmark of Six CNN Architectures †

**DOI:** 10.3390/diagnostics16182960

**Published:** 2026-09-13

**Authors:** Jia-Lun Huang, Chun-Yuan Lin, Chia-Wei Lin, Charles C. N. Wang

**Affiliations:** 1 Ph.D. Program in Artificial Intelligence, College of Information and Electrical Engineering, Asia University, 500 Lioufeng Rd., Wufeng District, Taichung 413, Taiwan; 013495@tool.caaumed.org.tw (J.-L.H.);; 2 Department of Emergency Medicine, China Medical University Hospital, No. 2, Yude Rd., North District, Taichung 404, Taiwan; 3 School of Medicine, College of Medicine, China Medical University, No. 91, Hsueh-Shih Rd., North District, Taichung 404, Taiwan; 4 Department of Computer Science and Information Engineering, Asia University, 500 Lioufeng Rd., Wufeng District, Taichung 413, Taiwan; 5 Department of Bioinformatics and Medical Engineering, Asia University, 500 Lioufeng Rd., Wufeng District, Taichung 413, Taiwan; 6 Center for Precision Health Research, Asia University, 500 Lioufeng Rd., Wufeng District, Taichung 413, Taiwan

**Keywords:** acute cholecystitis, contrast-enhanced computed tomography, deep learning, convolutional neural network, transfer learning, benchmarking, computer-aided diagnosis, emergency radiology, medical image analysis

## Abstract

**Background/Objectives:** Acute cholecystitis requires timely diagnosis, yet contrast-enhanced computed tomography (CT) interpretation can be challenging under a high radiology workload. This proof-of-concept study benchmarked convolutional neural network (CNN) architectures for slice-level classification, not a clinically validated diagnostic tool. **Methods:** Contrast-enhanced CT from 80 patients (51 acute cholecystitis, 29 controls) at a single center (2018–2020) yielded 1060 annotated axial slices. Acute cholecystitis was defined by a Tokyo Guidelines 2018–based composite reference standard. All slices from a patient were assigned to one partition only. Six ImageNet-pretrained architectures were fine-tuned: ResNet-18/50/101, Inception-V3, MobileNet-V3-small/large. Group comparisons used Student’s *t* test, chi-square with Yates’ correction, or Fisher’s exact test (R version 4.0.3; Python/SciPy). **Results:** On the internal held-out test set (213 slices; 122 acute-cholecystitis slices, 91 control slices), ResNet-101 achieved the highest accuracy (0.915) and specificity (0.857), while MobileNet-V3-large achieved the highest area under the curve (AUC) (0.97) and tied for the highest sensitivity (0.959) with far fewer parameters; across models, accuracy ranged from 0.812 to 0.915 and AUC from 0.89 to 0.97. **Conclusions:** Compact architectures showed competitive slice-level performance in this single-center benchmark. Because evaluation was slice-level in a small cohort with non-inflamed controls, these findings do not represent patient-level diagnostic accuracy. Patient-level aggregation, matched sensitivity analyses, explainability assessment, and external validation are required before clinical application.

## 1. Introduction

Acute cholecystitis, most commonly caused by obstruction of the cystic duct by gallstones, is a frequent gastrointestinal emergency requiring prompt diagnosis and treatment and a common complication of gallstone disease, one of the most prevalent gastrointestinal disorders worldwide [1,2,3,4]. In the USA, acute cholecystitis accounts for more than 200,000 hospital admissions per year [5] and contributes to the substantial burden of gastrointestinal disease [6]. Delayed or inaccurate diagnosis may lead to serious complications, including gallbladder perforation, abscess formation, sepsis, and increased mortality [7].

Contrast-enhanced computed tomography (CT) is widely used in the evaluation of suspected acute cholecystitis, particularly in emergency settings where abdominal pain may have a broad differential diagnosis and complications must be rapidly assessed [8,9]. CT can depict gallbladder wall thickening, gallbladder distention, pericholecystic fluid, inflammatory fat stranding, and alternative abdominal emergencies. Ultrasonography is commonly used as the first-line imaging modality, although reported diagnostic accuracy for acute cholecystitis varies across studies [10]. CT interpretation can also be challenging in early disease, atypical presentation, comorbid conditions, or complex anatomic settings. Increasing CT utilization has also increased radiologist workload, which may contribute to reporting delays and diagnostic variability [11].

Artificial intelligence (AI), particularly deep learning, has increasingly been applied to medical image analysis. Convolutional neural networks (CNNs) can automatically learn hierarchical image features from raw pixel data and have demonstrated strong performance in disease classification, lesion detection, and organ segmentation across radiography, CT, magnetic resonance imaging, and ultrasound [12,13,14,15,16,17]. In selected imaging tasks, CNN-based models have reached diagnostic performance comparable to expert clinicians, although clinical translation remains limited by data quality, study design, reporting completeness, and external validation [18,19,20].

To date, relatively few studies have applied deep learning specifically to gallbladder inflammation. On CT, Okuda et al. trained a CNN to identify gangrenous cholecystitis in a two-institution cohort and reported diagnostic performance exceeding that of experienced reviewers [21]. Also on CT, Yang et al. developed a fully automated pipeline combining VGG-16–based gallbladder localization with U-Net segmentation to identify acute cholecystitis requiring cholecystectomy in a cohort of 250 cases and 270 controls [22]. Much of the remaining work has used ultrasound rather than CT; Yu et al., for example, used a ResNet-50–based detector to localize gallstones and the lightweight MobileNet-V2 to classify cholecystitis on point-of-care ultrasound images [23]. In a recent multicenter study, Chen et al. developed a deep learning model for acute cholecystitis on noncontrast CT in a three-center cohort with external validation and gradient-weighted class activation mapping for interpretability [24]. Despite these advances, the application of CNN-based deep learning to acute cholecystitis detection from contrast-enhanced CT remains relatively underexplored, and the relative performance of different CNN architectures for this task has not been systematically compared under a common experimental setting. This gap is clinically relevant because acute cholecystitis is common, time-sensitive, and frequently encountered in emergency departments. A reliable AI-assisted model could be evaluated as a second reader and for prioritization of high-risk CT examinations; whether such use would shorten the interval between emergency-department arrival and surgical consultation would need to be tested directly. In addition, lightweight CNN architectures are of particular interest in this setting because emergency departments often operate with constrained computational resources and stringent patient-privacy requirements, which may make on-premises inference preferable to cloud-based processing; the present study, however, did not evaluate deployment feasibility.

In this study, we developed and evaluated multiple transfer learning-based CNN architectures for slice-level classification of acute cholecystitis on contrast-enhanced abdominal CT images. We compared the performance of ResNet, Inception-V3, and MobileNet-V3 variants using an internal held-out test set. The objective was to assess the feasibility and comparative performance of these models as a step toward potential future clinical evaluation of AI-assisted CT interpretation. Although the patient cohort was modest, the study was designed as an exploratory proof-of-concept investigation aimed at comparing CNN architectures under a controlled, leakage-controlled setting rather than at establishing definitive clinical performance estimates. The specific contribution of this work is therefore methodological rather than clinical: rather than proposing a new model, it evaluates six representative architectures head-to-head on the same cohort, using a harmonized preprocessing framework with architecture-specific input resolutions, identical augmentation procedures, and architecture-specific batch sizes and training durations. No formal superiority or equivalence testing between architectures was performed. This design allows the performance-versus-parameter-count trade-off between deep residual networks and lightweight mobile architectures to be described on the same cohort under a harmonized pipeline with architecture-specific settings, providing a controlled architectural benchmark against which subsequent patient-level and multicenter studies can be positioned.

## 2. Materials and Methods

### 2.1. Study Design, Setting, and Ethical Approval

This retrospective, single-center diagnostic model development study used contrast-enhanced abdominal CT examinations retrieved from the picture archiving and communication system of China Medical University Hospital, Taiwan. The study period was January 2018 to December 2020. The study protocol was approved by the Research Ethics Committee of China Medical University Hospital (protocol code CMUH114-REC3-068, approved on 15 April 2025). The requirement for informed consent was waived because of the retrospective design and use of anonymized imaging data.

### 2.2. Patient Cohort and Reference Standard

The study included 80 patients, comprising 51 patients with acute cholecystitis (mean age 62.7 ± 17.8 years; 22 men, 43.1%) and 29 controls without acute cholecystitis (mean age 44.9 ± 15.6 years; 10 men, 34.5%). In total, 80 contrast-enhanced CT examinations (one per patient) were evaluated. The final image dataset contained 1060 axial CT image slices, including 580 slices from patients with acute cholecystitis and 480 slices from controls without acute cholecystitis. Acute cholecystitis was confirmed using a composite clinical reference standard applying the Tokyo Guidelines 2018 (TG18) diagnostic criteria [25], which combine local signs of inflammation, systemic signs of inflammation, and characteristic imaging findings. Of the 51 patients with acute cholecystitis, 44 (86.3%) were surgically and/or pathologically confirmed; the remaining 7 (13.7%) did not undergo cholecystectomy and were classified on the basis of the TG18 clinical and imaging criteria alone. Controls were selected from patients who underwent abdominal CT for indications other than biliary disease and showed no clinical or imaging evidence of acute cholecystitis. Asymptomatic cholelithiasis was not an exclusion criterion and was present in three control patients.

**Figure 1:**
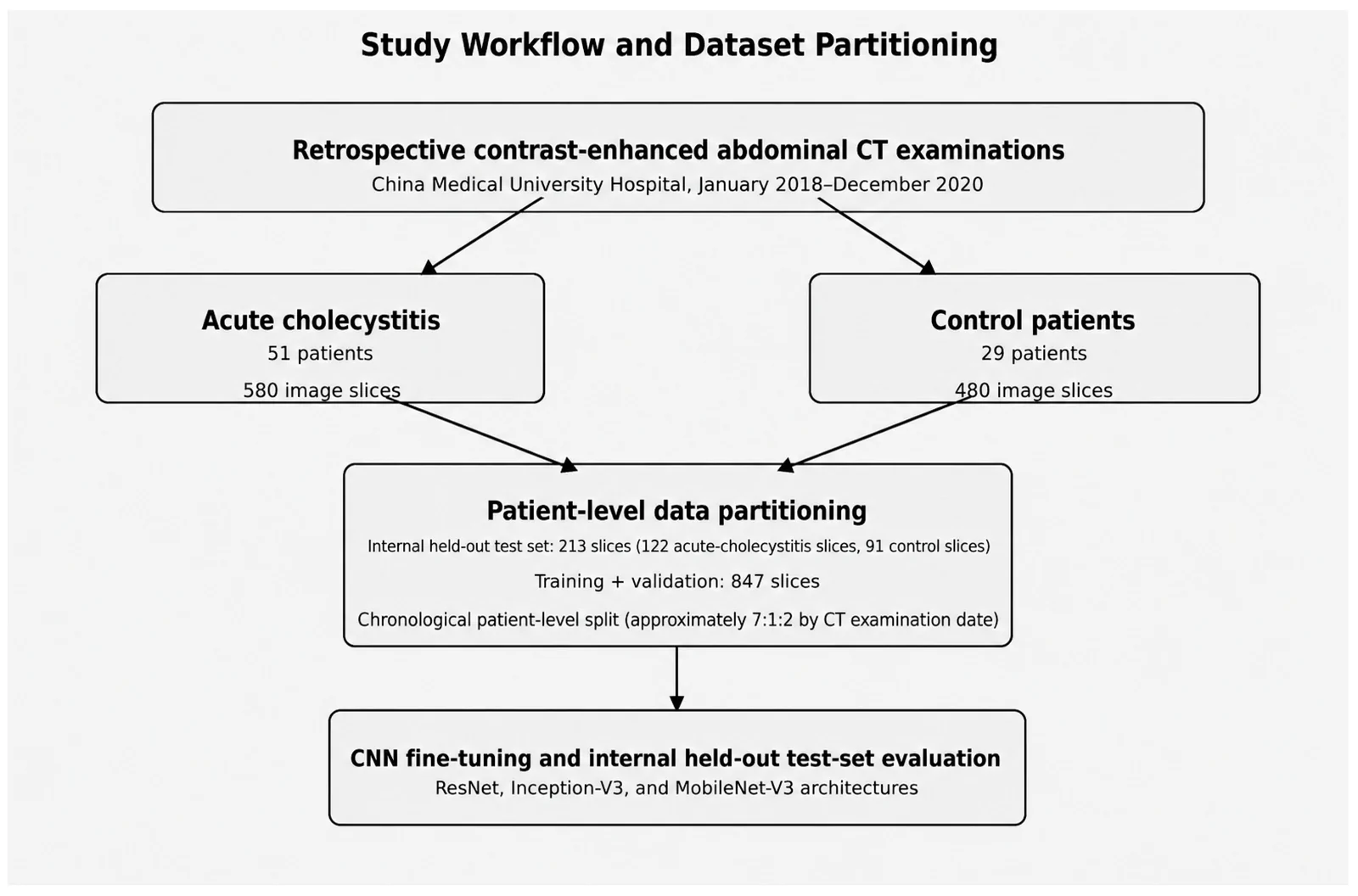
Study workflow and patient-level dataset partitioning.

Multiple axial slices were selected from each volumetric CT examination to capture gallbladder and pericholecystic regions relevant to diagnosis. To reduce information leakage, all CT image slices from the same patient were assigned exclusively to a single partition: training, validation, or testing (Figure 1). The dataset was divided at the patient level in an approximately 7:1:2 ratio of training, validation, and test data. Partitioning was performed chronologically rather than at random: patients were ordered by the date of their CT examination, and the earliest patients were assigned to the training set, the next consecutive patients to the validation set, and the most recent patients to the internal held-out test set. This temporal partitioning ensures that no patient contributes slices to more than one partition and that the held-out evaluation is performed on patients imaged later than those used for model development. The partitioning rule and the resulting slice-level partition sizes were retained in the study records; the identities and the exact number of patients within each partition were not, as noted in Section 2.6. Of the 1060 slices, 213 (122 acute-cholecystitis slices and 91 control slices) were held out as the internal held-out test set, and the remaining 847 slices formed the training and validation partitions. Because slices were partitioned at the patient level, the realized test-set size differed slightly from an exact ratio-based split.

Predefined exclusion criteria were applied to maintain diagnostic clarity. Patients were not included if they had undergone prior cholecystectomy, had chronic cholecystitis without an acute component, had known or suspected gallbladder malignancy, had examinations with substantial motion or beam-hardening artifact precluding evaluation, or had incomplete clinical records that prevented reliable diagnostic verification.

### 2.3. Data Variables and Data Management

The following variables were extracted from the hospital electronic medical record for each patient: age, sex, body mass index, presence of diabetes mellitus, presence of gallstones, the final diagnosis, and whether surgical or pathological confirmation was obtained. Imaging variables comprised the CT acquisition date, scanner and protocol information, and the number of axial slices selected per examination. Clinical and demographic variables were compiled and organized in Microsoft Excel (Microsoft Corporation, Redmond, WA, USA), and the resulting dataset was linked to the de-identified image files through a study-specific identifier. DICOM de-identification was performed using the pydicom library in Python, with patient identifiers removed from the DICOM headers before the images entered the analysis pipeline. Statistical analysis software is described in Section 2.7.

### 2.4. CT Image Annotation Procedure

The annotation process followed a multistage review strategy. Two experienced emergency physicians, each with more than 10 years of clinical experience, performed the initial image review and annotation while blinded to clinical and laboratory data. Radiological features considered relevant to acute cholecystitis included gallbladder wall thickening greater than 3 mm, pericholecystic fluid or inflammation, gallbladder distention, and gallstones when present.

The initial annotations were then verified in a single multidisciplinary stage by a panel comprising a gastroenterologist, a surgeon, and a radiologist, which confirmed the final labeling. Discrepancies between initial reviewers were resolved by consensus discussion. Regions of interest covering the gallbladder and associated inflammatory findings were manually annotated to guide slice selection and to verify that each selected slice contained gallbladder-relevant anatomy. The annotated regions of interest were used only for slice eligibility verification; the deep learning models received the corresponding full axial slices as input rather than cropped regions of interest, so that anatomical context surrounding the gallbladder was preserved during classification.

### 2.5. Image Preprocessing and Data Augmentation

Contrast-enhanced abdominal CT examinations were acquired on clinical multi-detector CT scanners using standard institutional protocols. CT acquisition parameters and contrast doses varied according to scanner type, patient body habitus, and routine clinical protocols. Tube voltage ranged from 80 to 120 kVp depending on the scanning protocol and patient size, with automatic tube-current modulation. Nonionic iodinated contrast medium containing 300–370 mg iodine/mL was administered intravenously at 70–100 mL or according to body weight, at an injection rate of 3.0–5.0 mL/s. Portal venous-phase images were reconstructed at a slice thickness of 5 mm for model development and evaluation. Scanner manufacturer and detector configuration were not recorded in a form that could be verified retrospectively and are therefore not reported; where a protocol parameter could not be reliably confirmed from the retained records, it has been omitted rather than stated as an unverified value. Portal venous-phase axial images were exported in DICOM format and de-identified as described in Section 2.3 before any image processing. The same contrast phase (portal venous-phase) was used uniformly across both the acute cholecystitis and control groups to prevent the models from learning phase-specific imaging characteristics rather than disease-related features.

All CT images were processed using a standardized pipeline. Images were retained at a matrix size of 512 × 512 pixels. Pixel values were normalized to a 0–1 range using min-max scaling; whether the scaling was computed per slice or across the dataset could not be confirmed from the retained records. No Hounsfield-unit windowing was applied; the pixel intensity values were normalized to a 0–1 range by min-max scaling as described above. Each single-channel CT slice was then replicated across three channels to match the input format expected by the ImageNet-pretrained backbones and resized to 224 × 224 pixels for ResNet-18, ResNet-50, ResNet-101, MobileNet-V3-small, and MobileNet-V3-large, or to 299 × 299 pixels for Inception-V3, corresponding to each network’s native input resolution. Channel-wise normalization was then applied to all six torchvision classification backbones using mean values of [0.471, 0.448, 0.408] and standard deviations of [0.234, 0.239, 0.242]. These correspond to COCO dataset statistics rather than to the ImageNet statistics conventionally paired with torchvision ImageNet-pretrained models; the same normalization was applied identically to all six architectures, so all models were compared under a harmonized preprocessing pipeline; we cannot exclude, however, that individual architectures differ in their sensitivity to this normalization mismatch.

Data augmentation was applied to the training set only, with transformations sampled independently at each training epoch (random horizontal flipping, random vertical flipping, and random rotation within the range of −180° to 180°), exposing the models to diverse training views in order to improve robustness and reduce overfitting, while leaving the validation and test sets unaltered.

### 2.6. CNN Architectures and Model Training

Six CNN model configurations were evaluated: ResNet-18, ResNet-50, ResNet-101, Inception-V3, MobileNet-V3-small, and MobileNet-V3-large [26,27,28]. Transfer learning was used by initializing models with ImageNet-pretrained weights [29,30]. Final classification layers were adapted for binary classification of acute cholecystitis versus control (no acute cholecystitis).

The evaluated architectures represent three distinct design philosophies. The residual networks (ResNet-18, ResNet-50, and ResNet-101) employ identity skip connections that stabilize gradient flow through deep networks and mitigate the vanishing-gradient problem; the three variants differ principally in depth and parameter count (approximately 11.7, 25.6, and 44.5 million parameters, respectively), enabling a controlled comparison of the effect of model capacity.

Inception-V3 (approximately 27.2 million parameters) uses multi-scale convolutional blocks that apply parallel filters of different sizes within a single layer, allowing the network to capture features across spatial scales. MobileNet-V3-small and MobileNet-V3-large (approximately 2.5 and 5.5 million parameters, respectively) employ depthwise-separable convolutions [31], squeeze-and-excitation blocks, and hardware-aware design to achieve competitive accuracy at substantially reduced computational cost; deployment characteristics on clinical hardware were not evaluated in this study. The principal design characteristics and parameter counts of the six evaluated architectures are summarized in Table 1.

Models were trained using stochastic gradient descent (SGD) with a learning rate of 0.01 and a momentum of 0.9. Batch size and the number of training epochs were configured per architecture: Inception-V3 (batch size 16, 100 epochs), MobileNet-V3-large (32, 100), MobileNet-V3-small (32, 100), ResNet-18 (32, 100), ResNet-50 (16, 150), and ResNet-101 (16, 100). The final model weights were selected according to the best validation performance before evaluation on the internal held-out test set. Several training details could not be reconstructed from the retained records and are therefore not reported rather than stated approximately: the specific validation metric used for checkpoint selection, the decision threshold applied to the model outputs, the random seed, and whether weight decay, a learning-rate scheduler, or early stopping were used. Similarly, the exact number of patients within each partition could not be recovered, and only the overall cohort composition and slice-level partition sizes are reported.

All models were fine-tuned end-to-end with all layers trainable, using cross-entropy loss. Experiments were implemented in PyTorch 1.10 and run on an NVIDIA RTX A5000 GPU (NVIDIA Corporation, Santa Clara, CA, USA).

### 2.7. Model Evaluation and Statistical Analysis

Model performance was evaluated on the internal held-out test set using accuracy, sensitivity, specificity, positive and negative predictive values, F1 score, false positive rate, false negative rate, and AUC. For accuracy, sensitivity, specificity, false positive rate, false negative rate, and the positive and negative predictive values, which are proportions, 95% descriptive confidence intervals, which do not account for within-patient correlation among slices, were calculated using the Wilson score method with the corresponding denominators (the full test set for accuracy, the number of acute cholecystitis slices for sensitivity and false negative rate, the number of control slices for specificity and false positive rate, and the number of slices predicted positive or negative for the positive and negative predictive values, respectively). F1 scores and AUC values are reported as point estimates obtained from the original model evaluation. Because per-slice predicted probabilities in a format suitable for bootstrap or DeLong-based interval estimation were no longer available for any of the six models, AUC confidence intervals and formal pairwise comparisons were not computed and are reserved for future external validation. Statistical analyses and metric computations were performed using R (version 4.0.3; R Foundation for Statistical Computing, Vienna, Austria) and Python. The baseline group comparisons reported in Table 2 were subsequently re-verified in Python using SciPy and NumPy; the verification script is provided as Supplementary Materials. Because the continuous variables are recomputed from the rounded summary statistics as published, re-computation reproduced all reported *p* values to within 0.001. Continuous variables were compared using the independent-sample Student’s *t* test, and categorical variables using the chi-square test with Yates’ continuity correction, with Fisher’s exact test applied when any expected cell count was below five.

For clarity, 95% confidence intervals are reported for the count-based proportions (accuracy, sensitivity, specificity, predictive values, and error rates), which can be derived from the retained confusion matrices. Confidence intervals for AUC could not be estimated because the continuous prediction scores were not retained. Cluster-adjusted intervals for F1 and for the other metrics could not be estimated because patient-linked slice-level predictions were unavailable; the intervals reported here are therefore naive slice-level Wilson intervals that treat each slice as an independent observation and are likely to understate the true uncertainty. This distinction is stated in the footnote to Table 3. The primary model evaluation was performed at the image-slice level. Because clinical diagnosis is ultimately patient-based, the absence of independent patient-level probability aggregation is acknowledged as an important limitation and a priority for future validation.

Calibration analyses, such as the Brier score, expected calibration error, and calibration curves, were not performed in this study.

### 2.8. Reporting Standards

The reporting of this AI-based diagnostic imaging study was aligned, where applicable, with relevant elements of the Checklist for Artificial Intelligence in Medical Imaging (CLAIM) 2024 update and the STARD-AI reporting guideline for diagnostic accuracy studies using artificial intelligence [32,33]. Because this study was retrospective and based on an internal dataset, several items related to prospective deployment and external validation could not be fully addressed.

## 3. Results

### 3.1. Patient Characteristics

The study population included 80 patients, corresponding to 80 contrast-enhanced CT examinations (one per patient), with 51 acute cholecystitis cases and 29 controls without acute cholecystitis. Patients with acute cholecystitis were older than controls (mean age, 62.67 ± 17.81 years vs. 44.90 ± 15.63 years; *p* < 0.001). Gallstones and diabetes were more frequent in the cholecystitis group. Body mass index did not differ significantly between groups. Patient characteristics are summarized in Table 2.

### 3.2. Dataset Partitioning

The dataset contained 1060 contrast-enhanced CT slices. The data were divided at the patient level in an approximately 7:1:2 ratio of training, validation, and test data, with partitions assigned chronologically by CT examination date as described in Section 2.2. The internal held-out test set comprised 213 slices (122 acute-cholecystitis slices and 91 control slices), and the remaining 847 slices formed the training and validation partitions. The patient-level assignment strategy prevented slices from the same patient appearing across multiple partitions.

### 3.3. Model Performance

**Figure 2:**
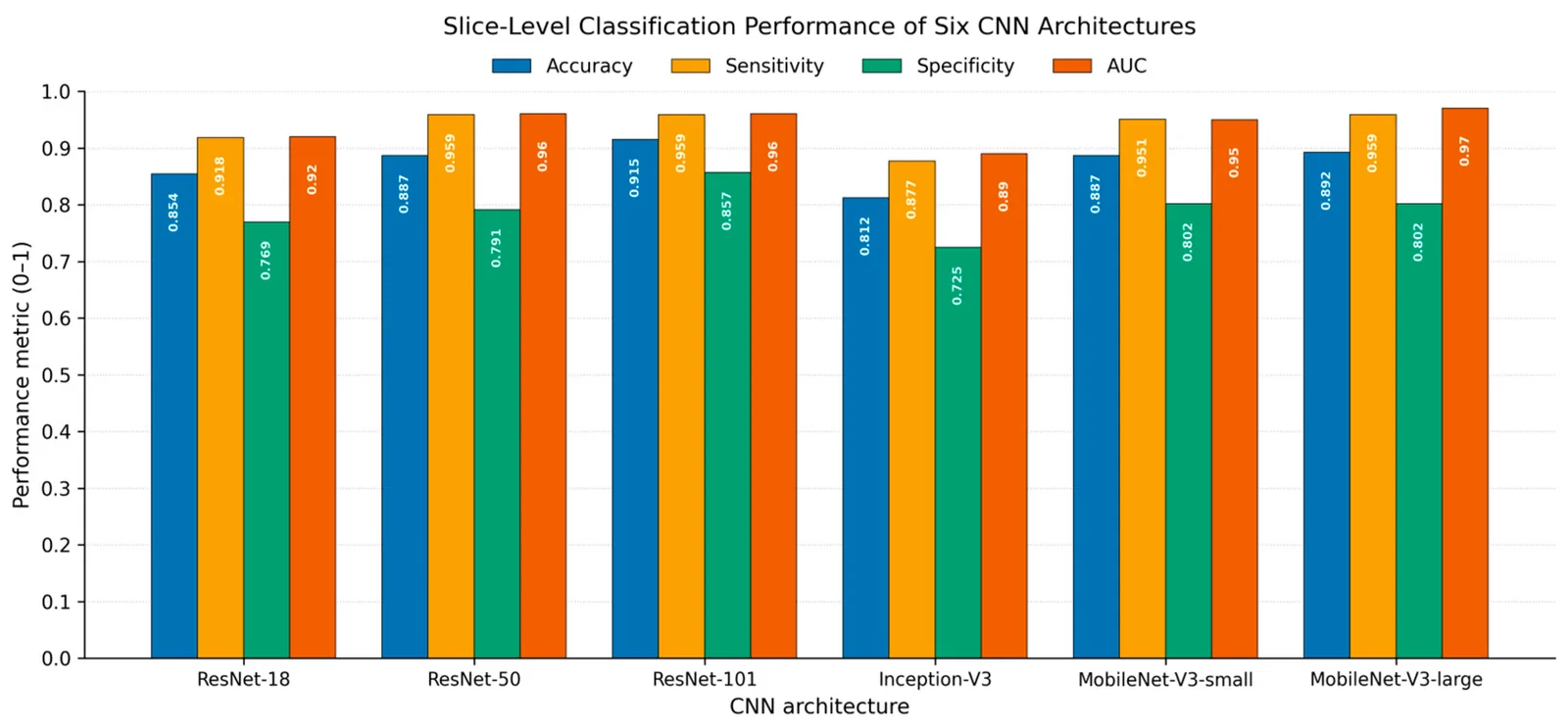
Slice-level classification performance of the six evaluated CNN architectures on the internal held-out test set, shown as a grouped bar chart of accuracy, sensitivity, specificity, and AUC. ResNet-101 achieved the highest accuracy and specificity, whereas the lightweight MobileNet-V3-large achieved the highest AUC and tied for the highest sensitivity; Inception-V3 had the lowest point estimates across the four displayed metrics. Sensitivity exceeded specificity for every model. Wilson 95% confidence intervals are available only for the count-based proportions; F1 scores and AUC values are reported as point estimates. For the full set of metrics and the corresponding confusion matrices, see Table 3 and Table 4.

Performance metrics for the CNN models on the internal held-out test set are shown in Table 3, with the corresponding confusion matrices in Table 4 and the overall comparison in Figure 2. Two architectures stood out. ResNet-101 achieved the highest overall accuracy (0.915; 95% CI, 0.870–0.946) and the highest specificity (0.857; 95% CI, 0.771–0.915), correctly classifying 117 of 122 acute-cholecystitis slices and 78 of 91 control slices. MobileNet-V3-large, a lightweight architecture, achieved the highest AUC (0.97) and shared the highest sensitivity (0.959; 95% CI, 0.908–0.982) and lowest false negative rate (0.041) with ResNet-50 and ResNet-101, while requiring far fewer parameters than the residual networks. ResNet-50 and MobileNet-V3-small yielded intermediate point estimates (accuracy 0.887 for both; AUC 0.96 and 0.95, respectively). ResNet-18 reached an accuracy of 0.854 (95% CI, 0.801–0.896) and an AUC of 0.92, and Inception-V3 had the lowest observed point estimates, with the lowest accuracy (0.812; 95% CI, 0.754–0.859), lowest specificity (0.725), highest false positive rate (0.275), and lowest AUC (0.89). Across all six models, sensitivity (0.877–0.959) was consistently higher than specificity (0.725–0.857), indicating that false positives among control slices were the main source of error. AUC values are reported as point estimates; confidence-interval estimation and formal pairwise comparison of AUCs are reserved for future external multicenter validation on independent patient-level outcomes.

### 3.4. Literature Context

**Figure 3:**
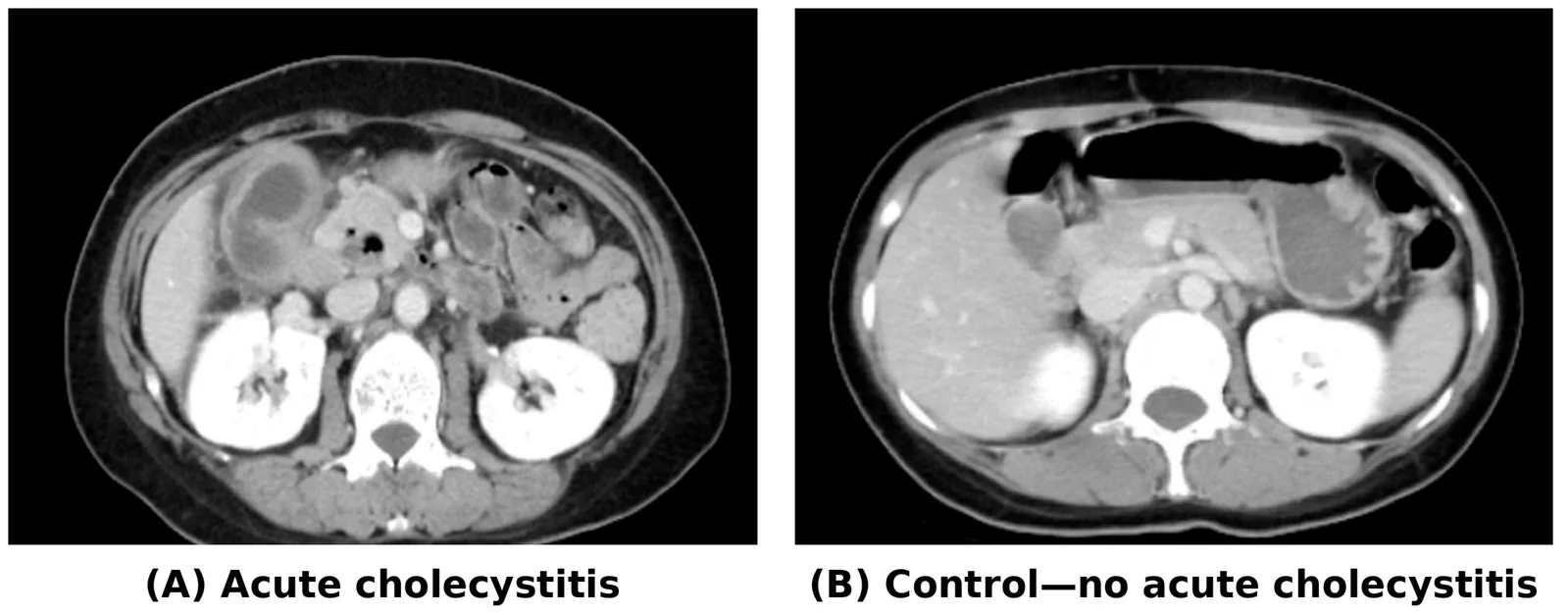
Representative contrast-enhanced CT images. (**A**) Acute cholecystitis with gallbladder wall thickening and pericholecystic inflammatory change. (**B**) Gallbladder without inflammatory change in a control CT image (control group: no acute cholecystitis).

For broader contextual reference, the diagnostic performance reported in this study can be considered alongside published literature on imaging performance for the diagnosis of acute cholecystitis. In the 2012 systematic review and meta-analysis by Kiewiet et al. [8], only one CT study met the inclusion criteria, reporting a sensitivity of 94% (95% CI: 73%, 99%) at a specificity of 59% (95% CI: 42%, 74%), and the authors concluded that CT was at that time still underevaluated. More recent evidence gives a substantially higher estimate of CT specificity: in a 2024 systematic review and meta-analysis of head-to-head studies, CT had a pooled sensitivity of 83.9% (95% CI: 78.4%, 88.2%) and a pooled specificity of 94.0% (95% CI: 82.0%, 98.0%) [34]. Measured against this more current benchmark, the slice-level specificity of the best-performing model in the present study (0.857 for ResNet-101) remains below contemporary estimates of radiologist CT interpretation, and no model exceeded it. These comparisons are in any case not directly interpretable, because they involve different datasets, reading conditions, reference standards, and units of analysis; patient-level reader-versus-AI studies would be required for a meaningful comparison. Representative contrast-enhanced CT images of an acute cholecystitis case and a control are shown in Figure 3.

## 4. Discussion

### 4.1. Principal Findings

This study demonstrates the feasibility of CNN-based models for slice-level classification of acute cholecystitis on selected contrast-enhanced CT images. These findings must, however, be read in light of two features of the study design that are addressed in detail below: the control group comprised predominantly non-inflamed gallbladders rather than the acute abdominal differential encountered in emergency practice (appendicitis, pancreatitis, diverticulitis, and biliary colic were not represented, and uncomplicated cholelithiasis was severely under-represented, being present in only 3 of 29 controls), and the case and control groups differed in age, diabetes prevalence, and gallstone prevalence. Together these introduce spectrum-effect and confounding biases from non-representative control selection, and we cannot exclude the possibility that the models exploited age- or gallstone-related signals rather than inflammatory imaging features, and the performance estimates reported here are therefore likely to be optimistic and may overestimate real-world performance. Among the evaluated architectures, ResNet-101 achieved the highest accuracy and specificity, while the lightweight MobileNet-V3-large achieved the highest AUC and tied for the highest sensitivity with substantially fewer parameters. These findings support further evaluation of residual and lightweight architectures for slice-level CT classification under patient-level, matched, and external validation settings.

### 4.2. Clinical and Diagnostic Significance

From a diagnostic imaging perspective, this study evaluates the transformation of contrast-enhanced CT image data into a classification output through a controlled model development pipeline. If subsequently validated at the patient level and in external prospective cohorts, models of this type could be evaluated for second-reader or examination-prioritization workflows in emergency radiology. These applications were not assessed in the present study: no workflow, turnaround-time, or patient-outcome measures were collected, no prospective assessment was undertaken, and the model was not applied to unselected examinations. Any effect on preoperative management therefore remains hypothetical and would require prospective, patient-level evaluation.

We position the resulting system as a research-stage image-classification model for emergency radiology workflows rather than a definitive diagnostic standard, intended, if validated, to supplement rather than replace expert review; no prioritization or safety benefit, and no effect on radiologist attention or on the timing of surgical consultation, was evaluated in this study.

The relatively compact MobileNet-V3-large architecture, with approximately 5.5 million parameters and depthwise-separable convolutions, is identified here as a candidate for future deployment benchmarking. Inference time, memory consumption, model size, and workstation-level performance were not measured in the present study, no deployment scenario was evaluated, and these would need to be benchmarked directly before any deployment claim could be supported. In principle, such a lightweight model could run on local hospital hardware, which is relevant to privacy-preserving, low-latency deployment; however, these deployment scenarios are hypothetical at this stage and were not evaluated in the present study.

### 4.3. Architecture-Level Interpretation

Because per-slice probability outputs were not retained, no formal pairwise statistical comparison between architectures could be performed; the orderings described below are numerical differences observed within this single experiment and should not be read as demonstrated superiority of one architecture over another. The pattern of performance across architectures is nonetheless informative. Among the residual networks, the deepest variant, ResNet-101, achieved the highest accuracy and specificity, whereas the shallowest, ResNet-18, performed worst within the ResNet family; within the present dataset and training configuration, the deeper ResNet-101 achieved better test-set accuracy and specificity than the shallower ResNet variants. At the same time, the lightweight MobileNet-V3-large yielded the highest AUC and tied for the highest sensitivity, with an accuracy of 0.892 and a specificity of 0.802, while its standard ImageNet-1K implementation has approximately 88% fewer reference parameters than that of ResNet-101 (5.5 versus 44.5 million); it did not, however, exceed ResNet-101 in accuracy or specificity. This combination of discriminative performance and low parameter count is a notable observation of the present study, because the observed slice-level point estimates spanned a narrow range across architectures differing substantially in parameter count; no equivalence testing was performed, so these architectures cannot be described as equivalent. MobileNet-V3-large combines high slice-level sensitivity and AUC with a substantially lower parameter count, which may provide a promising architectural foundation for future edge-oriented benchmarking. This should not be read as an established deployment capability: inference time, memory use, model size, and workstation-level performance were not measured in this study. Inception-V3 had the lowest values for all higher-is-better metrics and the highest false positive and false negative rates. The reason for this cannot be determined from the present study and warrants further investigation. A consistent pattern across all six models was that sensitivity exceeded specificity, indicating that the models were more prone to false positives on control slices than to missing acute cholecystitis; this operating pattern is one in which false positives are more frequent than missed disease; whether such a profile is clinically acceptable was not evaluated here and would require threshold-specific utility and workflow assessment.

### 4.4. Relation to Prior Work

Deep learning has shown substantial promise in multiple medical imaging tasks, including chest radiography, dermatology, and abdominal imaging [12,13,14,15,16,17,18,19,20,35,36,37,38]. However, systematic reviews have emphasized that many AI imaging studies remain limited by retrospective design, small datasets, incomplete reporting, and lack of external validation [35,36,37]. The present study contributes to this literature by focusing on acute cholecystitis, a clinically important emergency abdominal condition that has received comparatively less attention in CT-based AI research. A recent multicenter study demonstrated accurate deep learning–based diagnosis of acute cholecystitis and prediction of suppuration using noncontrast CT, including external validation and Grad-CAM interpretability [24]. Our work provides a controlled head-to-head evaluation of multiple ImageNet-pretrained CNN architectures for contrast-enhanced CT–based acute cholecystitis classification using patient-level data partitioning, with an emphasis on evaluating lightweight architectures as candidates for future on-premises deployment benchmarking. Whereas Chen et al. [24] leveraged Grad-CAM to interpret noncontrast CT features, the present study focuses on establishing a controlled architectural comparison for contrast-enhanced workflows; integrating multi-scale interpretability methods adapted to contrast-enhanced anatomy is a natural and important direction for future work. Our findings should therefore be regarded as complementary to, rather than competing with, prior noncontrast CT-based clinical validation work: Chen et al. established clinical feasibility on noncontrast CT with external validation and multimodal integration, whereas the present study provides a contrast-enhanced CT-specific architectural benchmark under patient-level partitioning and identifies lightweight architectures as candidates for future deployment benchmarking.

### 4.5. Trustworthiness, Safety, and Clinical Translation

Although the observed performance was high, clinical translation requires evidence beyond internal slice-level test performance. A trustworthy AI system for acute cholecystitis should demonstrate robust performance across scanners, acquisition protocols, institutions, disease severity levels, and patient subgroups.

The present results should therefore be interpreted as evidence of feasibility rather than readiness for autonomous clinical use. Prospective integration studies are needed to evaluate whether such models can reduce diagnostic delay, improve radiology workflow, or affect patient-centered outcomes.

### 4.6. Limitations

Several limitations must be acknowledged. First, this was a retrospective single-center study with a relatively small patient cohort, which may limit generalizability. Although 1060 image slices were analyzed, the full cohort comprised 80 patients (51 with acute cholecystitis and 29 controls), and the exact number of patients in the held-out test partition could not be recovered. The effective independent sample size underlying the reported test metrics was therefore smaller than 80 and cannot be stated precisely, which may have contributed to optimistic performance estimates. No a priori statistical power calculation was performed, and a cohort of this size cannot support precise estimation of diagnostic performance or reliable detection of differences between architectures; the reported metrics should therefore be read as descriptive comparisons within a single controlled experiment rather than as generalizable performance estimates. Second, although patient-level partitioning was used to reduce leakage, model evaluation was conducted at the CT slice level rather than using independent patient-level aggregation. Adjacent slices from the same CT volume are correlated, and future studies should aggregate slice-level predictions into patient-level diagnoses using aggregation strategies such as majority voting, probability averaging, or multiple-instance learning approaches. Slice-level evaluation was adopted deliberately in the present study because its primary aim was to compare the representational capacity of different CNN backbones for this slice-level classification task on selected gallbladder-centered images; evaluating all architectures on the same per-slice basis provides a controlled and directly comparable benchmark. We also note that partitioning was chronological rather than random, so the held-out evaluation was performed on patients imaged later than those used for model development; this provides a temporal hold-out, but it does not substitute for external validation at an independent center. We note that patient-level aggregation could in principle have been applied identically to every architecture and would not have precluded the comparison; it was not performed because the per-slice predictions paired with patient identifiers are no longer available. The present study therefore evaluates slice-level discrimination only.

Because multiple adjacent slices originate from the same CT examination and share anatomical continuity, slice-level evaluation may overestimate real-world diagnostic performance relative to fully independent patient-level testing. For the proportion-based metrics, we report 95% confidence intervals using the Wilson score method; however, because slices from the same patient are not statistically independent, these slice-level intervals may be narrower than intervals that properly account for within-patient correlation. Because per-slice predicted probabilities paired with patient identifiers were no longer available for any of the six models in this retrospective benchmark, joint cluster-adjusted modeling, such as generalized estimating equations or patient-level cluster bootstrapping, could not be performed; the reported intervals should therefore be interpreted as descriptive measures of slice-level classification variance under a controlled benchmarking setting rather than as generalized clinical precision estimates. Formal patient-level uncertainty estimation would require newly collected or recovered data and is therefore deferred to future external multicenter validation. The observed test performance should be interpreted as encouraging but preliminary, and may partly reflect the controlled dataset composition and the slice-based evaluation setting; performance on independent multicenter cohorts may differ and could be lower. Because the effective statistical unit is the patient rather than the CT slice, the reported slice-level results should be regarded as a measure of image-classification capability rather than direct clinical diagnostic accuracy, and future studies should report patient-level AUROC, calibration, and clinical utility metrics using independent cohorts.

Third, the control group was drawn from 29 patients without acute inflammatory gallbladder changes on CT (three of whom had asymptomatic cholelithiasis), which simplifies the binary classification task relative to real emergency-department practice where patients may present with pancreatitis, appendicitis, or other acute abdominal conditions. The acute cholecystitis and control groups also differed substantially in age (62.7 versus 44.9 years; *p* < 0.001) and in gallstone prevalence (60.8% versus 10.3%; *p* < 0.001), and the models may therefore have partially learned age-related degenerative features, stone-related imaging patterns, or other cohort-related signals rather than inflammatory features alone. It should nevertheless be noted that the multistage annotation protocol explicitly defined gallbladder wall thickening greater than 3 mm and pericholecystic fluid or inflammation as core diagnostic features during expert review, so that slice selection and eligibility verification were guided by active inflammatory imaging features rather than by chronological age or the mere presence of gallstones. However, because the models received the corresponding full axial slices rather than the annotated regions, this annotation procedure does not establish which image features drove the predictions. Because the TG18 reference standard incorporates imaging findings, and the analyzed slices were selected on the basis of gallbladder-related imaging features, the labeling process was not fully independent of the index test. This concern is partly mitigated because 44 of 51 patients (86.3%) had operative and/or pathological corroboration; however, the degree of independence from the diagnostic CT interpretation cannot be fully established retrospectively, since the decision to operate may itself have been informed by the preoperative imaging. The concern remains greatest for the 7 patients classified using Tokyo Guidelines 2018 clinical and imaging criteria without cholecystectomy. Separately, because the models received the full image rather than an annotated region, the labeling protocol does not constrain which image features they used, and it therefore cannot reduce the possibility that age-, gallstone-, or other covariate-related signals contributed to the predictions. Only a matched sensitivity analysis, stratified testing, or interpretability assessment could evaluate this, and none was feasible here. In particular, the present study did not include a separate cohort of patients with gallstones but without acute inflammation, which would have allowed a more rigorous test of whether the models can discriminate active cholecystitis from cholelithiasis alone. Because the image data and trained models used in this study are no longer accessible, such analyses cannot be performed retrospectively. Future studies using newly collected or recovered data should include a targeted sensitivity analysis restricted to age-matched controls and to gallstone-bearing but non-inflamed gallbladders, together with patient-level cluster-adjusted estimation, to formally disentangle inflammatory signal from these confounders. Future work should include such intermediate cohorts together with patients with uncomplicated cholelithiasis, chronic cholecystitis, biliary colic, cholangitis, pancreatitis, appendicitis, and nonspecific abdominal pain as more challenging and clinically realistic differential diagnoses. Fourth, the study used selected gallbladder-centered CT slices, which does not fully replicate real-world whole-volume CT interpretation. The present model should therefore be viewed as an image-classification benchmark rather than a fully automated computer-aided detection system capable of localizing the gallbladder across whole abdominal CT volumes; such fully automated detection and classification pipelines should be developed and validated in future work.

Fifth, no external validation cohort was available. Without testing across institutions, scanners, contrast protocols, and patient populations, model transportability cannot be established. The augmentation scheme included unrestricted rotation and vertical flipping, which can produce anatomically implausible orientations for axial CT; this choice could not be revisited in the present analysis and should be reassessed in future model development. Finally, model explainability and calibration analyses were not included in this version of the study. Future work should include Grad-CAM or related methods, calibration curves, Brier scores, and decision curve analysis to support clinical interpretability and risk-informed deployment.

### 4.7. Future Directions

Future research should prioritize multicenter external validation, prospective patient-level evaluation, and integration of imaging with clinical and laboratory variables. Multimodal models combining CT images, white blood cell count, C-reactive protein, liver function tests, fever status, and right upper quadrant tenderness may better approximate real-world diagnostic reasoning. Lightweight model deployment, automated whole-volume gallbladder localization, and prospective workflow studies should also be pursued before clinical implementation.

Probability calibration should also be evaluated in future work, using reliability diagrams, the Brier score, and the expected calibration error, because highly discriminative models may still produce poorly calibrated confidence estimates in clinical deployment settings. Decision-support tools for emergency radiology require not only correct ranking of cases but also trustworthy probability magnitudes; complementary analyses such as decision curve analysis may further clarify the net clinical benefit at relevant operating thresholds.

## 5. Conclusions

CNN-based deep learning models demonstrated promising slice-level classification performance for acute cholecystitis detection from contrast-enhanced CT images in this retrospective single-center study. ResNet-101 achieved the highest accuracy, while the lightweight MobileNet-V3-large achieved the highest AUC and tied for the highest sensitivity while using substantially fewer parameters than ResNet-101. These findings are hypothesis-generating: they support further evaluation of lightweight and residual CNN architectures for slice-level CT classification but do not establish patient-level diagnostic accuracy or clinical deployment readiness; multicenter, patient-level validation, matched sensitivity analyses, explainability analysis, and prospective workflow assessment are required before any clinical application.

## Figures and Tables

**Table 1:** Design characteristics and reference parameter counts of the six evaluated CNN architectures.

Architecture	Design Family	Reference Parameter Count of the Standard ImageNet-1K Model (M)	Key Design Feature
ResNet-18	Residual network	11.7	Identity skip connections (shallow depth)
ResNet-50	Residual network	25.6	Identity skip connections with bottleneck blocks
ResNet-101	Residual network	44.5	Identity skip connections (deep)
Inception-V3	Inception	27.2	Multi-scale parallel filters within layers
MobileNet-V3-small	Lightweight	2.5	Depthwise-separable conv + SE block + hardware-aware
MobileNet-V3-large	Lightweight	5.5	Depthwise-separable conv + SE block + hardware-aware

Note: Parameter counts are reference values for the standard torchvision ImageNet-1K implementations, rounded to one decimal place and including the auxiliary classifier where applicable. The classifier head was replaced for binary classification, so the parameter counts of the fitted models differ from these reference values; the exact fitted-model counts were not directly recoverable. SE = squeeze-and-excitation.

**Table 2:** Characteristics of the study population.

Characteristic	Cholecystitis (*n* = 51)	Control (*n* = 29)	*p* Value
Age, years, mean ± SD	62.67 ± 17.81	44.90 ± 15.63	<0.001
BMI, kg/m^2^, mean ± SD	24.39 ± 3.80	23.28 ± 3.57	0.204
Male sex, *n* (%)	22 (43.1%)	10 (34.5%)	0.602
Diabetes, *n* (%)	11 (21.6%)	0 (0.0%)	0.006
Gallstones, *n* (%)	31 (60.8%)	3 (10.3%)	<0.001

Note: Data are presented as mean ± standard deviation for continuous variables and as *n* (%) for categorical variables. Continuous variables were compared using the independent-samples Student’s *t* test; categorical variables were compared using the chi-square test with Yates’ continuity correction, with Fisher’s exact test used when any expected cell count was less than five. BMI, body mass index.

**Table 3:** Slice-level performance metrics of the six CNN architectures on the internal held-out test set.

Metric	ResNet-18	ResNet-50	ResNet-101	Inception-V3	MobileNet-V3-Small	MobileNet-V3-Large
Accuracy	0.854 (0.801–0.896)	0.887 (0.838–0.923)	0.915 (0.870–0.946)	0.812 (0.754–0.859)	0.887 (0.838–0.923)	0.892 (0.843–0.927)
Sensitivity	0.918 (0.856–0.955)	0.959 (0.908–0.982)	0.959 (0.908–0.982)	0.877 (0.807–0.924)	0.951 (0.897–0.977)	0.959 (0.908–0.982)
Specificity	0.769 (0.673–0.844)	0.791 (0.697–0.862)	0.857 (0.771–0.915)	0.725 (0.626–0.806)	0.802 (0.709–0.871)	0.802 (0.709–0.871)
False positive rate	0.231 (0.156–0.327)	0.209 (0.138–0.303)	0.143 (0.085–0.229)	0.275 (0.194–0.374)	0.198 (0.129–0.291)	0.198 (0.129–0.291)
False negative rate	0.082 (0.045–0.144)	0.041 (0.018–0.092)	0.041 (0.018–0.092)	0.123 (0.076–0.193)	0.049 (0.023–0.103)	0.041 (0.018–0.092)
Positive predictive value	0.842 (0.771–0.894)	0.860 (0.792–0.909)	0.900 (0.836–0.941)	0.811 (0.735–0.868)	0.866 (0.798–0.913)	0.867 (0.799–0.914)
Negative predictive value	0.875 (0.785–0.931)	0.935 (0.857–0.972)	0.940 (0.867–0.974)	0.815 (0.717–0.884)	0.924 (0.844–0.965)	0.936 (0.859–0.972)
F1 score (positive)	0.878	0.907	0.929	0.843	0.906	0.911
F1 score (negative)	0.819	0.857	0.897	0.767	0.859	0.864
AUC	0.92	0.96	0.96	0.89	0.95	0.97

Note: Accuracy, sensitivity, specificity, false positive rate, false negative rate, and positive and negative predictive values are reported as point estimates with 95% confidence intervals calculated using the Wilson score method on the internal held-out test set (*n* = 213; 122 acute-cholecystitis slices and 91 control slices). These are naive slice-level intervals: they treat slices as independent observations and may be too narrow, because within-patient correlation among slices was not modeled. F1 scores are reported as point estimates because the Wilson method applies to a single binomial proportion whereas F1 combines two proportions with different denominators; no interval was computed for it. AUC values are reported as point estimates; confidence intervals and formal pairwise comparison of AUCs were not computed in the present internal evaluation and are reserved for future external validation. The positive class refers to acute cholecystitis and the negative class refers to controls without acute cholecystitis.

**Table 4:** Confusion matrices of the six CNN models on the internal held-out test set.

Model	TP	FN	TN	FP
ResNet-18	112	10	70	21
ResNet-50	117	5	72	19
ResNet-101	117	5	78	13
Inception-V3	107	15	66	25
MobileNet-V3-small	116	6	73	18
MobileNet-V3-large	117	5	73	18

Note: TP = true positive (acute cholecystitis correctly classified); FN = false negative; TN = true negative (control correctly classified); FP = false positive. The internal held-out test set comprised 122 acute-cholecystitis slices and 91 control slices (total *n* = 213). For each model, TP + FN = 122 and TN + FP = 91.

## Data Availability

The de-identified image dataset, the trained model weights, and the per-slice prediction outputs generated for this study are no longer accessible to the study team and cannot be shared. The data were not publicly deposited because of privacy and ethical restrictions applying to patient imaging data. The point estimates and confusion matrices reported here were generated during the original analysis period, before the source data became inaccessible; the Wilson confidence intervals and the Python/SciPy re-verification of Table 2 were computed subsequently from those retained summary results. The representative images shown in Figure 3 were prepared and exported during the original analysis period. The script used to re-verify the baseline statistical comparisons reported in Table 2 is provided as Supplementary Materials.

## Supplementary Materials

The following supporting information can be downloaded at: https://www.mdpi.com/article/10.3390/diagnostics16182960/s1, Supplementary File S1. Table S1—Baseline characteristics: statistical tests (table2_statistics_scipy.py).
